# Pembrolizumab Alone or Combined With Chemotherapy in Advanced NSCLC With PD-L1 ≥50%: Results of a Retrospective Study

**DOI:** 10.3389/fonc.2021.691519

**Published:** 2021-06-28

**Authors:** Ya Chen, Yanan Wang, Zhengyu Yang, Minjuan Hu, Yanwei Zhang, Fangfei Qian, Wei Zhang, Bo Zhang, Baohui Han

**Affiliations:** Department of Pulmonary, Shanghai Chest Hospital, Shanghai Jiao Tong University, Shanghai, China

**Keywords:** non small cell lung cancer, pembrolizumab, chemotherapy, immunotherapy, programed cell death protein 1 (PD-1)

## Abstract

**Objectives:**

Pembrolizumab plus platinum-based chemotherapy and pembrolizumab monotherapy (PM) both become standard of care in patients with advanced non-small-cell lung cancer (NSCLC) and a programmed death ligand 1 (PD-L1) tumor proportion score (TPS) greater than 50%. This study aimed to figure out the better treatment choice.

**Method:**

In this retrospective analysis, we compared the clinical efficacy of PM and PC as first-line treatment in NSCLC patients with a PD-L1 ≥50% and negative for genomic alterations in the EGFR and ALK genes.

**Result:**

Among the population, 115 patients received PC, and 91 patients received PM. Up to Dec 30, 2020, median follow-up was 17.13 months. The median progression-free survival (PFS) rates of PC and PM were 12.37 and 9.60 months (HR: 0.44, p < 0.001), respectively. The median overall survival (OS) rates were NE and 28.91 months (HR: 0.40, p = 0.005), respectively. Subgroup analysis found that the PFS benefit of PC was evident in most subgroups excepting patients with brain metastasis. The 1-year overall survival rates of PC and PM were 89.3% and 76.1%, respectively. The ORR was 61.7 and 46.9% (p = 0.004), respectively.

**Conclusion:**

In patients with previously untreated, PD-L1 ≥50%, advanced NSCLC without EGFR or ALK mutations, the addition of pembrolizumab to standard platinum-based chemotherapy seems to be the preferred treatment, which needs to be validated by further prospective trials.

## Introduction

The use of immune checkpoint inhibitors has greatly altered the standard of care in patients with advanced NSCLC. Pembrolizumab, an IgG4 monoclonal antibody against programmed cell death protein 1 (PD-1) has become a powerful treatment option in clinical practice nowadays.

The KEYNOTE-024 study compared pembrolizumab monotherapy (PM) *versus* chemotherapy in treatment-naïve patients with advanced non-small-cell lung cancer (NSCLC) with programmed death ligand 1 (PD-L1) tumor proportion score (TPS) of 50% or greater. PM achieved a remarkable improvement in terms of progression-free survival [PFS; hazard ratio (HR), 0.50; 95% CI, 0.37 to 0.68] and overall survival (OS; HR, 0.63; 95% CI, 0.47 to 0.86) (1, 2). Single-agent pembrolizumab becomes the standard of care in treatment-naïve NSCLC patients with a PD-L1 TPS ≥50% building on the results of KEYNOTE-024 study. Meanwhile, KEYNOTE-189 and KEYNOTE-407 revealed that pembrolizumab plus platinum-based chemotherapy (PC) significantly improved survival outcomes compared with chemotherapy in patients with metastatic non-squamous and squamous non-small-cell lung cancer (NSCLC), respectively.

Recently, updated analysis of KEYNOTE-024 showed that PM continued to provide remarkable clinical outcomes. The median PFS and OS of the PM group were 10.0 and 30.0 months, respectively. The 2-year overall survival rate was 51.5%, which was a breakthrough for NSCLC patients without EGFR/ALK mutation (1). Meanwhile, updated analysis of KEYNOTE-189 demonstrated that the PFS and OS of patients with PD-L1 ≥50% in the PC group were 11.1 and were not reached. The 2-year overall survival rate was 51.9% (3). Of interest, in patients with PD-L1 ≥50%, there seemed to be not much difference of the median PFS and the 2-year overall survival rate between the PM group in KEYNOTE-024 and the PC group in KEYNOTE-189. Recently, Liang et al. conducted an indirect comparison of clinical outcomes between immunotherapy plus chemotherapy (I + C) and immunotherapy alone. They found that I + C was superior to immunotherapy alone in terms of PFS (HR 0.54, 0.35–0.82) in patients with PD-L1 ≥50%. But the PFS benefit did not translate into an OS benefit (HR 0.75, 95% CI 0.51–1.10) (4). Kim et al. also compared the efficacy of I + C treatment and immunotherapy alone indirectly but reported different results. They found that pembrolizumab plus chemotherapy was superior to pembrolizumab alone in terms of PFS and OS in patients with PD-L1 ≥50% (5).

At present, PC and PM are both recommended with high evidence quality in NSCLC patients with PD-L1 TPS ≥50% without EGFR/ALK alterations according to NCCN and ASCO guideline (6). Of note, PM has been given a high-priority rating though it is difficult to figure out which one was the best option due to the absence of direct comparison. Several meta-analyses compared the efficacy of PC and PM indirectly but presented paradoxical result, which might be due to the inherent limitation such as the risk of systematic bias and confounding factors (5, 7–9). In this context, the present study aimed to figure out which therapy was the priority in this specific population by head-to-head comparison.

## Material and Methods

### Patients

The medical records of advanced NSCLC patients who received immunotherapy at the Shanghai Chest Hospital between Dec 1, 2017 and Oct 30, 2020 were screened. Two hundred and six patients met the following eligibility criteria: (1) advanced NSCLC (IIIB–IV); (2) histologically or cytologically proven NSCLC; (3) PD-L1 TPS ≥50% without sensitizing EGFR or ALK mutations; (4) pembrolizumab monotherapy or combined with chemotherapy as first-line treatment (chemotherapy agents were mainly pemetrexed, paclitaxel, or gemcitabine in combination with platinum) following standard medical instructions; (5) Eastern Cooperative Oncology Group performance status (ECOG PS) 0–1. Therapeutic schedule was decided by a physician under the principle that PC was priority, provided that patients has a high symptom and/or disease burden and/or large-volume visceral tumor and/or symptomatic brain metastasis (6). This study was approved by the Institutional Review Board of Shanghai Chest Hospital and carried out in accordance with the declaration of Helsinki.

### Programmed Death Ligand 1 Tumor Proportion Score and Gene Detection

Tumor samples were obtained by tissue biopsy at the time that disease was diagnosed. PD-L1 expression was assessed before treatment detected by the PD-L1 IHC 22C3 pharmDx assay. Expression was classified to several types according to the tumor proportion score, TPS <0, 1–49 and ≥50%. The amplification refractory mutation system (ARMS) was used as the routine molecular technique for EGFR detection following the protocol of the DxS EGFR mutation test kit. The immunohistochemistry (IHC) and break-apart fluorescence *in situ* hybridization (FISH) were used as the routine molecular technique for ALK rearrangement detection.

### Treatment and Clinical Response Evaluation

Therapeutic response evaluation, including enhanced chest computed tomography (CT) scan, and abdominal ultrasound scan, was performed every 4–6 weeks, while enhanced brain magnetic resonance imaging (MRI) was performed every half year if no lesion at baseline and no symptoms thereafter. If patients developed symptom during the treatment, the corresponding examination and evaluation were performed immediately. Clinical stage was determined by the 8th edition of the International Association for the Study of Lung Cancer (IASLC) tumor-node-metastasis (TNM) classification. The response was evaluated according to the Response Evaluation Criteria in Solid Tumors (RECIST) version 1.1.

### Statistical Analysis

The characteristics of patients were compared using the χ2 test for categorical variables. The primary endpoints were PFS (calculated from disease diagnosis to disease progression or the last follow-up), OS (calculated from disease diagnosis to death or the last follow-up), and ORR. The median PFS and OS were estimated using the Kaplan–Meier method and compared by the log-rank test. Hazard ratios and associated 95% confidence intervals were calculated with the use of a stratified Cox proportional-hazards model. All p values were two-sided, and a P value <0.05 was considered statistically significant. All statistical analyses were performed using SPSS version 22.0 (IBM Corporation, Armonk, NY, USA).

## Results

### Clinical Features

**Figure 1:**
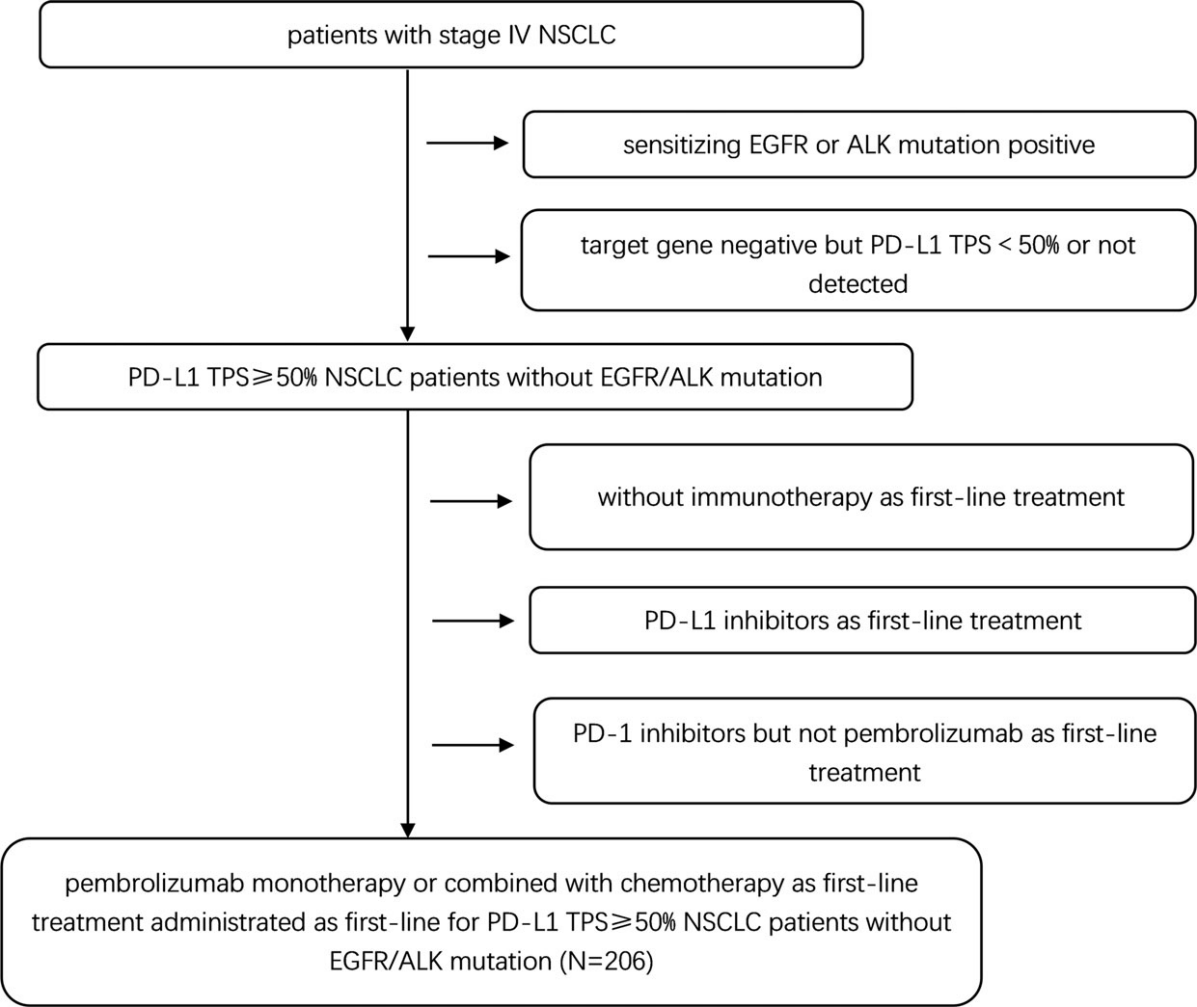
Patient selection flow-chart.

Two hundred and six patients met the eligibility criteria were included in this study. The patient selection procedure is shown in **Figure 1**. Among the 206 patients, 115 (55.8%) received PC and 91 (44.2%) received PM. The median follow-up time was 17.13 months. The median age was 65 (range 37–76) years and 67 (range 29–87) years in the PC and PM groups, respectively. Most patients were male (88.7% in PC and 87.9% in PM), current or former smoker (74.8% in PC and 79.1% in PM), stage IV (63.5% in PC and 64.8% in PM), adenocarcinoma (64.3% in PC and 53.8% in PM) and without brain metastasis (80.0% in PC and 90.1% in PM). The patients’ baseline demographic and disease characteristics were generally well balanced between PC and PM groups except more patients with brain metastasis (BM) were in the PC group (p = 0.047) (**Table 1**).

**Table 1:** Clinical characteristic of 206 patients of advanced NSCLC.

Variable	Pembrolizumab plus chemotherapy (N = 115)	Pembrolizumab (N = 91)	P value
Age			
Median (range)—year	65 (37–76)	67 (29–87)	
<65years—no. (%)	53 (46.1)	32 (35.2)	0.465
Sex—no. (%)			0.489
Male	102 (88.7)	80 (87.9)	
Female	13 (11.3)	11 (12.1)	
Smoking			0.529
Current or former smoker	86 (74.8)	72 (79.1)	
Never smoker	29 (25.2)	19 (20.9)	
Stage			0.840
IIIB–IIIC	42 (36.5)	32 (35.2)	
IV	73 (63.5)	59 (64.8)	
Histology			0.127
Squamous	41 (35.7)	42 (46.2)	
Adenocarcinoma	74 (64.3)	49 (53.8)	
Extrapulmonary metastasis			0.717
NO	59 (51.3)	49 (53.8)	
YES	56 (48.7)	42 (46.2)	
Central nervous system metastasis			0.047
NO	92 (80.0)	82 (90.1)	
YES	23 (20.0)	9 (9.9)	
Previous therapy for non-metastatic disease, n (%)			
Thoracic radiotherapy	19 (16.5)	13 (14.3)	0.660
Adjuvant therapy	9 (7.8)	9 (9.9)	0.602

Data are median (range) or n (%). NSCLC, non-small-cell lung cancer.

### Survival Analysis of PC and PM as First-Line Treatment in Patients With Advanced NSCLC

**Figure 2:**
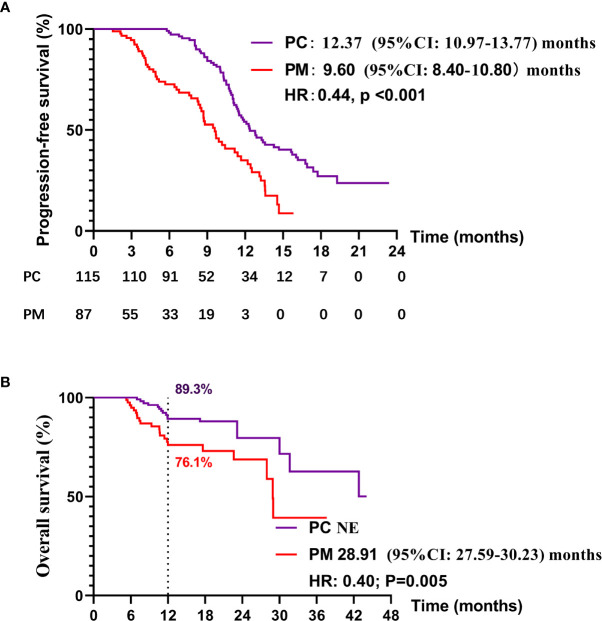
Kaplan–Meier curves for **(A)** progression-free survival (PFS) and **(B)** overall survival (OS) in PC and PM groups.

Up to Dec 30, 2020, 69 of 115 patients (60.0%) in the PC group and 57 of 91 patients (56.0%) in PM group had disease progression on first-line treatment. One hundred of 115 patients (87.0%) in the PC group and 69 of 91 patients (75.8%) in the PM group were still alive. The median PFS of PC and PM groups was 12.37 months (95% CI: 10.97–13.77) and 9.60 months (95% CI: 8.40–10.80), respectively (HR:0.44, p < 0.001) (**Figure 2A**). Median overall survival of PC and PM groups was not reached and 28.91 months (HR: 0.40, p = 0.005, **Figure 2B**), respectively. The 1-year overall survival rates of PC and PM were 89.3% and 76.1%, respectively.

### Subgroup Analysis of PFS

**Figure 3:**
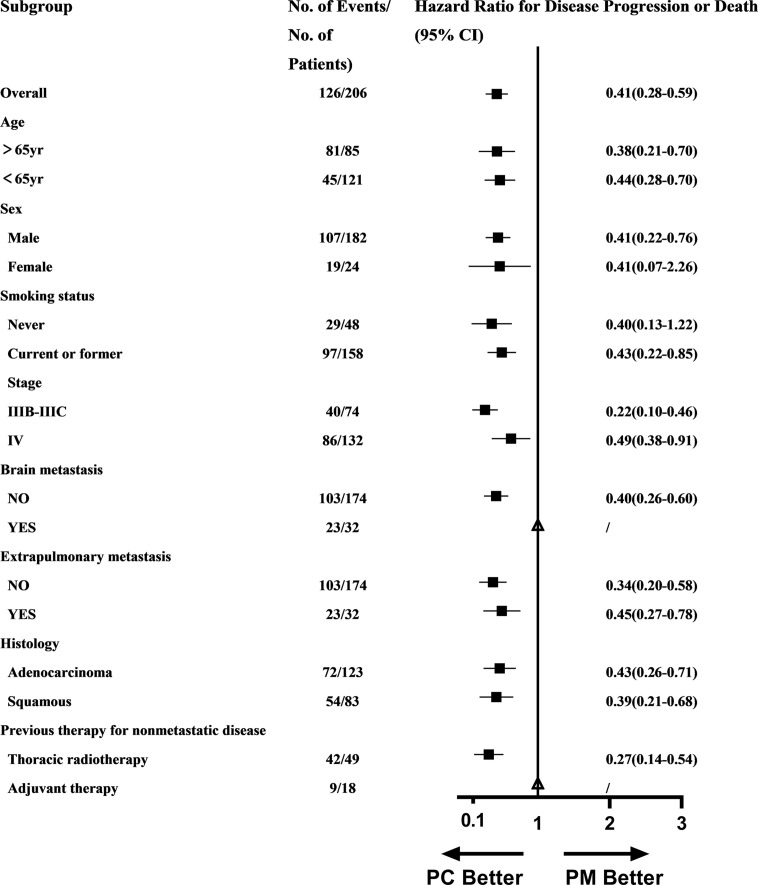
Subgroup analysis of PFS in PC and PM groups.

A PFS benefit with PC was evident in most subgroups assessed (**Figure 3**), except for patients with brain metastasis (could not be calculated due to small sample in the PM group) and patients with previous adjuvant therapy (could not be calculated due to small sample).

### Tumor Response of PC and PM as First-Line Treatment in Patients With Advanced NSCLC

**Figure 4:**
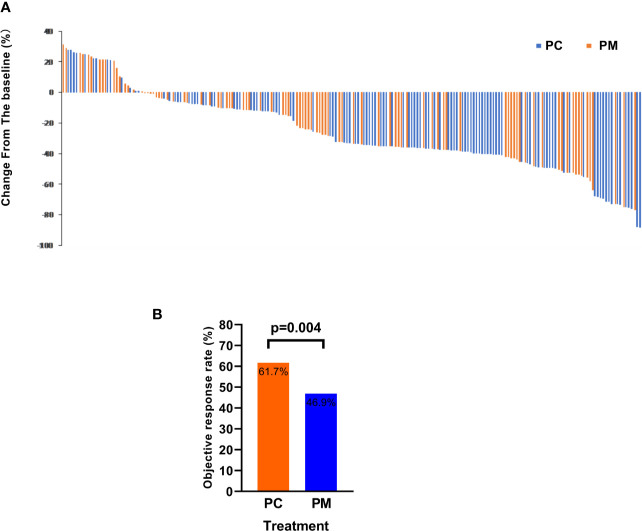
The objective response rate is shown as a percent change of target lesions from baseline in PC and PM groups **(A)** and histograms showing the response rate **(B)**.

The ORR (the proportion of patients with a confirmed complete or partial response) of PC and PM was 61.7 and 46.9% (p = 0.004), respectively. The disease control rates (the proportion of patients with a confirmed complete or partial response or stable disease) were 94.8 and 87.7%, respectively (p = 0.292). The change from baseline in the sum of the longest diameters of target lesions is shown in **Figures 4A, B**.

## Discussion

Survival analysis of PC and PM as first-line treatment in NSCLC patients with a PD-L1 TPS ≥50% was retrospectively investigated in the present study, which, to our knowledge, was analyzed directly for the first time.

Baseline characteristics were well balanced between PC and PM groups except more patients with brain metastasis were in the PC group. We found that adding chemotherapy to pembrolizumab resulted in a risk of disease progression that was 50% lower than the risks with pembrolizumab alone. Clinical outcomes, including PFS, OS, and ORR were improved significantly in the PC treatment arm.

KEYNOTE-189 (patients with adenocarcinoma) and KEYNOTE-407 (patients with squamous NSCLC) reported the median PFS as 11.1 and 8.00 months in patients with PD-L1 ≥50%, respectively (10, 11). The present study found the median PFS of PC group was 12.37 months, higher than the results of KEYNOTE-189 and KEYNOTE-407. The 1-year overall survival rate and ORR of PC in our study were 89.3% and 61.7%, which was comparable with the results of KEYNOTE-189 and KEYNOTE-407 in patients with PD-L1 ≥50% (11). Subgroup analysis of East Asia population and the rest of world population found that East Asia population had more reduced risk of disease progression and death than others in KEYNOTE-407. The HR of PFS was 0.49 and 0.58 in East Asia population and others, respectively. The HR of OS was 0.44 and 0.69, respectively. This racial difference might explain the greater survival benefit of PC in our study (11, 12). The median PFS of PM in our study was 9.60 months, which was between the values of 10.3 and 7.1 months of the patients with PD-L1 ≥50% in the KEYNOTE-024 and KEYNOTE-042 studies, respectively (2, 13). The median OS was 28.91 months in our study, slightly lower than 30.0 months of KEYNOTE-024 (1). ORR was 46.9% in our study, which was similar with the values of 44.8 and 39% in the KEYNOTE-024 and KEYNOTE-042 studies (2, 13). Those comparable results indicated the validity and reliability of our data. Recently, Wu et al. showed that Chinese patients from the KEYNOTE-042 global and China extension (NCT03850444) study could also obtain significant OS benefit from pembrolizumab treatment compared with standard chemotherapy (14). But details are not yet available. Thus, we look forward to explore whether efficacy of PM was comparable between our study and Wu’s study.

Current treatment choice in the first-line setting in patients with NSCLC without targetable gene alterations depends on the PD-L1 expression levels. PC is the only alternative treatment of patients with low PD-L1 TPS (<50%) while PC and PM are both standard of care of patients with high PD-L1 TPS ≥50%). Deciding the optimal treatment in patients with a PD-L1 level ≥50% remains a challenge nowadays due to no direct comparison between PC and PM. Patients with a PD-L1 TPS ≥50% in KEYNOTE-042 did not replicate the remarkable result of KEYNOTE-024 because PFS was not superior in the pembrolizumab group and, in fact, was below that seen with chemotherapy for the first 6 months of treatment (15). And the difference was not explained convincingly. Thus, whether chemotherapy is indispensable for NSCLC patients with PD-L1 TPS ≥50% needs to be explored (15).

Several meta-analyses focused on the comparison of PC and PM but presented paradoxical result. A meta-analysis compared the OS between PM and PC in RCT trials and found that PC showed significant superiority to PM (HR: 0.87; 95% CI: 0.79–0.95) in general patients (6). Liu et al. focused on patients with PD-L1 TPS ≥50% and found that PC was superior to PM in terms of OS (HR =0.74, 95% CI: 0.56–0.98) but there was no difference on PFS (HR =0.83, 95% CI: 0.53–1.3) (9). Nevertheless, another analysis revealed a result that was almost converse to Liu’s. PC performed significantly better than PM in terms of ORR (OR 1.60, 95% CI 1.20–2.20), PFS (HR 0.52, 95% CI 0.37–0.71) but not for OS (HR 0.75, 95% CI 0.51–1.10) in patients with PD-L1 high expression (10). In addition, Liang et al. found that PC was comparable to PM in terms of OS and PFS (HR = 1.01, 95% CI: 0.63 to 1.57 and HR = 0.59, 95% CI: 0.35 to 1.06) in patients with PD-L1 high expression by meta-analysis (7). Those results of meta-analysis were different or even opposite, which might be due to the inherent limitation such as the risk of systematic bias and confounding factors. Different search strategy, data extraction and statistical analysis also contributed to the huge difference. Thus, head-to-head comparison is needed to disperse the fog. Our study indicated that PC significantly improved PFS, OS, and ORR compared with PM. However, further clinical trials are needed to validate this benefit. INSIGNA (NCT 03793179), an ongoing randomized phase III study, compares the clinical outcomes of the pembrolizumab in combination with chemotherapy and pembrolizumab alone in treatment-naïve advanced non-squamous NSCLC with PD-L1 expression ≥1%. PERSEE (NCT 04547504), another ongoing phase III study, compares the pembrolizumab–chemotherapy combination and pembrolizumab alone as first-line treatment for advanced NSCLC with a PD-L1 expression ≥50%. These two ongoing trials focus on the same subject but target different populations, and we look forward to their clinical outcomes.

Subgroup analysis in our study found that the PFS benefit of PC was evident in most subgroups excepting for patients with brain metastasis and patients with previous adjuvant therapy because HR and 95% CI could not be calculated due to the small sample. Subgroup analysis may indicate that the benefit of PC over PM was universal across almost all population. Aguilar EJ et al. found that patients with NSCLC and PD-L1 TPS ≥90% treated with first-line pembrolizumab significantly improved clinical outcomes among patients with a PD-L1 TPS ≥50%, which indicated that PD-L1 expression can be divided more exquisitely according to prognosis (16). The subgroup of PD-L1 should also be further analyzed in our study but most PD-L1 expressions were labeled undefined, only with records of PD-L1 ≥50%. Aguilar EJ’s study reminded us that the refinement of PD-L1 expression level was needed.

Apart from PD-L1 expression, more predictive biomarkers or prognostic factors, including but not limited to blood-based tumor mutational burden (bTMB), high body mass index (BMI), lactate dehydrogenase level (LDH), lung immune prognostic index (LIPI), serine/threonine kinase 11 gene (STK11) mutation, STING pathway, should be further analyzed to help identify the most effective treatment regimens for this specific population (17–21).

Ferrara R et al. proposed that PD-1/PD-L1 inhibitors or single-agent chemotherapy might be associated with hyperprogressive disease (22). In our study, every patient in the PC group received platinum-based chemotherapy instead of single-agent chemotherapy. Further study needs to be conducted to explore whether platinum is indispensable for NSCLC patients receiving immunotherapy plus chemotherapy. The mechanism of chemotherapy plus immunotherapy is not fully understood. However, there was evidence suggesting that chemotherapy can stimulate the antigenicity and immunogenicity of the host by enhancing antigen processing and presentation and by eliminating immune-suppressive myeloid derived suppressor cells (MDSC) and regulatory T cells (Tregs) (7, 23–26). Meanwhile, Ramakrishnan et al. proposed that chemotherapy may stimulate tumor cells to CTLs *via* the upregulation of mannose-6-phosphate receptors (MPRs), and autophagy may exert a tremendous influence in the immunogenic signaling during chemotherapy, which might contribute to the synergistic effect of chemotherapy and immunotherapy (23). Further exploration of the mechanism is needed.

Our study is limited by its retrospective nature. First, the sample size was relatively small and was collected from one center. Second, selection bias existed inevitably because of the missing data. Though the baseline clinical characteristics of patients in PC and PM groups were balanced well, we recognized the existence of selection bias that patients with no significant medical comorbidities were more likely to receive PC while patients with no significant medical comorbidities were more likely to receive PM. Also, the follow-up time was relatively short so that the median OS of PC was not mature, but to some extent K–M curve had showed significant difference between the two groups. Further follow-up should be conducted to confirm the OS benefit. Finally, though our study found that the clinical efficacy of the combination group was better than monotherapy, adverse events were not compared because of the incomplete records. Thus, prospective trials (INSIGNA and PERSEE) are indispensable for validating both efficacy and adverse events of these two treatments.

## Conclusions

Direct comparison of clinical outcome between PC and PM in NSCLC patients with PD-L1 TPS ≥50% without driver alterations was reported for the first time. We found that PC improved PFS, OS, and ORR benefit over PM.

## Data Availability Statement

The datasets presented in this article are not readily available because of the following: ethical requirements for Shanghai chest hospital. Requests to access the datasets should be directed to 18930858216@163.com.

## Author Contributions

YC, YW, and ZY have substantial contributions to the conception or design of the work, the collection and analysis of data, the writing and editing of the article. The rest authors have given substantial contributions to the work by providing editing and writing assistance. All authors contributed to the article and approved the submitted version.

## Funding

This research was supported by Shanghai Xuhui District municipal health commission [grant number XHLHGG201806] and Shanghai Shenkang three-year project [grant number SHDC2020CR4017].

## Conflict of Interest

The authors declare that the research was conducted in the absence of any commercial or financial relationships that could be construed as a potential conflict of interest.
